# Primary mediastinal B-cell lymphoma: upfront high-dose chemotherapy with autologous stem cell transplantation results in favorable long-term outcome and may spare radiotherapy

**DOI:** 10.1007/s00277-026-07009-w

**Published:** 2026-04-17

**Authors:** Tino Vollmer, Tim Strüßmann, Franziska Glatzki, Konrad Aumann, Kristina Fritsch, Justus Duyster, Roland Mertelsmann, Jürgen Finke, Reinhard Marks

**Affiliations:** 1 Department of Hematology, Oncology and Stem Cell Transplantation, Freiburg University Medical Center, Hugstetter Straße 55, Freiburg, 79106 Germany; 2 Institute for Surgical Pathology, Faculty of Medicine, University of Freiburg, Freiburg, Germany; 3 Kantonsspital Baden, Baden, Switzerland

**Keywords:** Primary mediastinal B-cell lymphoma, Autologous stem cell transplantation, Dose-dense immuno-chemotherapy, Mediastinal radiotherapy

## Abstract

The optimal therapeutic approach for primary mediastinal B-cell lymphoma remains unknown and varies in clinical practice. Dose-adjusted EPOCH-R or R-CHOP-based immuno-chemotherapy with mediastinal radiotherapy are acceptable treatment options. We report a retrospective analysis of PMBL patients treated with high-dose chemotherapy (HDCT) and autologous stem cell transplantation (ASCT) consolidation or dose-dense immuno-chemotherapy alone. A comparison of the two consolidation strategies showed no significant difference in 5-year PFS (95.5% vs. 82.4%, HR 0.22 95% CI 0.03–1.69, *p* = 0.13) or OS (100% vs. 87.1%, HR 0.1 95% CI 0.006–1.69, *p* = 0.11), with a trend toward better survival in the preplanned HDCT/ASCT group. Notably, 35.3% of patients received additional HDCT/ASCT after an inadequate response to dose-dense immuno-chemotherapy. When comparing all patients, with or without first-line HDCT/ASCT consolidation, regardless of the initial consolidation strategy, we found a significant difference in 5-year PFS (96.2% vs. 75%, HR 0.09 95% CI 0.01–0.83, *p* = 0.03) and OS (100% vs. 80.1%, HR 0.03 95% CI 0.002–0.7, *p* = 0.03) for patients treated with HDCT/ASCT. In addition, mediastinal radiotherapy was administered significantly less frequently in patients treated with HDCT/ASCT. In conclusion, high-dose chemotherapy and autologous stem cell transplantation may be a valid strategy to induce a favorable long-term outcome and spare radiotherapy in patients with PMBL.

**Supplementary Information:**

The online version contains supplementary material available at 10.1007/s00277-026-07009-w.

## Introduction

Primary mediastinal large B‑cell lymphoma (PMBL) is now recognized as a distinct clinicopathologic entity with features overlapping diffuse large B‑cell lymphoma and classical nodular sclerosing Hodgkin lymphoma [1–4]. Patients are typically young adults, often women, who present with bulky, locally invasive anterior mediastinal masses and frequently have elevated lactate dehydrogenase, advanced stage disease and high‑intermediate or high age‑adjusted International Prognostic Index (aaIPI) scores [5–7]. Despite generally favorable biology, up‑front management remains challenging. In the pre‑rituximab era, dose‑intensified regimens improved outcomes compared with cyclophosphamide, doxorubicin, vincristine and prednisone (CHOP) but were usually combined with mediastinal radiotherapy (RT) [5–8]. Addition of rituximab (R) to standard CHOP resulted in superior PFS for good-risk patients in a subgroup analysis of the MabThera International Trial [9]. In the rituximab era, R‑CHOP‑based approaches and dose‑adjusted etoposide, prednisone, vincristine, cyclophosphamide and doxorubicin (DA EPOCH‑R) have both been widely adopted. DA‑EPOCH‑R has shown excellent long‑term results with very low RT utilization, but its superiority over dose‑dense R‑CHOP‑based therapy has not been established in randomized trials and mediastinal RT continues to be used in a substantial proportion of patients treated with standard regimens [9]. A large prospective study of the International Extranodal Lymphoma Study Group (IELSG) reported a 5-year PFS of 86% in patients treated with rituximab in combination with methotrexate (MTX) with leucovorin rescue, doxorubicin, cyclophosphamide, vincristine, prednisone, and bleomycin (MACOP-B) or etoposide, doxorubicin, cyclophosphamide, vincristine, prednisone and bleomycin (VACOP-B) followed by involved field radiotherapy [10]. Given the young age of many PMBL patients, the long‑term cardiopulmonary and second‑malignancy risks of mediastinal RT are of particular concern [11–13]. Our institution has used a dose‑dense R‑CHOP‑based regimen (R‑MTX‑CHOP‑14) as standard induction for PMBL, with consolidation strategies varying between planned HDCT/ASCT and dose‑dense immunochemotherapy with RT given at the physician’s discretion. We therefore conducted a retrospective analysis of consecutively treated patients to explore long‑term outcomes with these consolidation strategies, the impact of HDCT/ASCT and the possibility of reducing RT exposure.

## Methods

### Patients and eligibility

The Comprehensive Cancer Center Freiburg database was screened for all consecutive patients aged 18–65 years with de‑novo, biopsy‑proven PMBL treated between 2005 and 2022. The study cohort represents the entire eligible patient population during this period; there were no exclusion criteria other than primary central nervous system involvement. All available primary histology specimens were centrally reviewed by an experienced hematopathologist and confirmed as PMBL according to the WHO classification, including assessment of morphology and immunophenotype (CD23 and/or CD30).

### Staging procedures

Baseline evaluation included standard clinical assessment, laboratory tests, computed tomography imaging and bone marrow biopsy; additional procedures (for example pleural or cerebrospinal fluid cytology or flow cytometry) were performed when clinically indicated. Patients with relevant pericardial or pleural effusions were classified as advanced stage disease. End‑of‑treatment (EOT) response assessment was performed with FDG‑PET/CT whenever feasible and interpreted according to contemporary standardized criteria and Deauville scoring [14–16]. EOT response assessment was conducted 6–8 weeks after autologous transplant for patients receiving preplanned HDCT/ASCT or 6–8 weeks after dose-dense immuno-chemotherapy alone.

### Study design and treatment modalities

The patients received a dose‑dense R‑CHOP‑based induction protocol (R‑MTX‑CHOP‑14) as previously described [17]. For patients responding to induction, two consolidation strategies were used in routine practice: (i) planned consolidation with HDCT/ASCT, or (ii) continuation with dose‑dense immunochemotherapy alone, with mediastinal RT considered according to PET response and physician judgment. Some patients initially allocated to dose‑dense immunochemotherapy were escalated to HDCT/ASCT because of incomplete metabolic response. The detailed composition and dosing of R‑MTX‑CHOP‑14, salvage regimens used for stem cell mobilization, BEAM conditioning, transplant supportive care, anti‑infective prophylaxis, fertility preservation measures and toxicity grading are described in Supplementary Methods. Response was assessed by CT and/or FDG‑PET/CT 6–8 weeks after completion of planned therapy (after ASCT for transplant candidates or after immunochemotherapy for non‑transplant consolidation). Complete metabolic response (CMR) was defined as Deauville scores 1–3; scores 4–5 prompted consideration of treatment escalation and, where feasible, histologic confirmation.

### Statistical analysis

Statistical analyses were performed with SAS V9.2 and GraphPad Prism 10^®^. Time-point for data cut-off was 01st June 2024. PFS and OS rates were estimated using the Kaplan-Meier method. Survival distributions were compared using univariate Cox proportional hazards regression models. Hazard ratios (HR) were reported with accompanying two-sided 95% confidence intervals (CI). We performed Fisher’s exact test to detect differences between patients receiving HDCT/ASCT and without HDCT/ASCT. Multivariate Cox proportional hazards regression models were not performed due to the low number of events. Statistical significance was defined as *p* ≤ 0.05.

## Results

### Characteristics of the patients

We identified 41 consecutively treated PMBL patients. Median age was 33 years (range 22–63), and 65.9% were female. Most patients had good performance status (ECOG 0–1 in 78%), advanced stage disease (stage III/IV in 75.6%), bulky mediastinal masses (97.6%) and elevated LDH (92.7%). The majority of patients had high‑intermediate (56%) or high (22%) aaIPI scores. Detailed baseline characteristics are summarized in Table 1. Central pathology review confirmed PMBL in all 29 cases with available material; the remaining externally reported diagnoses were consistent with PMBL. One patient presented at relapse with features intermediate between PMBL and classical Hodgkin lymphoma (mediastinal grey zone lymphoma). Although clonality analysis was inconclusive, disease evolution from PMBL was clinically favored.

**Table 1:** Patient characteristics

	2005-2022
Total patients	41 (100%)
Sex	
Male	14 (34.1%)
Female	27 (65.9%)
Median age, y (range)	33 (22-63)
< 60 y	39 (95.1%)
> 60 y	2 (4.9%)
Performance status	
ECOG 0-1	32 (78%)
ECOG ≥ 2	9 (22%)
Disease stage	
I/II	10 (24.4%)
III/IV	31 (75.6%)
Bulky disease	40 (97.6%)
LDH elevated more than normal	38 (92.7%)
Extranodal sites	
≥ 2	11 (26.8%)
Bone-marrow involvement	0 (0%)
Age-adjusted IPI score	
Low-intermediate (1)	9 (22%)
High intermediate (2)	23 (56%)
High (3)	9 (22%)
Histology	
PMBL	41 (100%)

### Treatment modalities

All 41 patients received R‑MTX‑CHOP‑14 induction; one had primary refractory disease. Among the 40 responders, 23 were intended for upfront HDCT/ASCT consolidation and 17 for consolidation with dose‑dense immunochemotherapy alone. Planned HDCT/ASCT was delivered in 95.6% of patients in that group; one patient declined ASCT and received RT instead. In the dose‑dense immunochemotherapy cohort, 6 of 17 patients (35.3%) received no further consolidation, 5 (29.4%) underwent HDCT/ASCT because of insufficient response, and 5 (29.4%) received consolidative mediastinal RT. Overall, 24 of 40 responders (60%) ultimately underwent HDCT/ASCT, and 10 (25%) received mediastinal RT as part of first‑line management (Table 2 and Supplementary Fig. 1).

**Table 2:** Consolidation treatment for PR/CR patients

	All patients	Preplanned HDCT/ASCT	Dose-dense immuno-chemotherapy
	No. (%)	No. (%)	No. (%)
All patients	40 (100%)	23 (100%)	17 (100%)
No consolidation treatment	6 (15%)	-	6 (35.3%)
HDCT/ASCT	24 (60%)	19 (82.7%)	5 (29.4%)
HDCT/ASCT + RT	4 (10%)	3 (13%)	1 (5.9%)
RT	6 (15%)	1 (4.3%)	5 (29.4%)

### Toxicity

No treatment‑related deaths occurred during first‑line therapy, and no secondary malignancies were observed during follow‑up. As expected, grade 3–4 hematologic toxicities were common: neutropenia in 87.5%, thrombocytopenia in 70% and anemia in 65% of patients; grade 3 febrile neutropenia occurred in 65%. Grade 3–4 non‑hematologic organ toxicities were less frequent and reversible. Hematologic toxicity and febrile neutropenia were more common in patients undergoing HDCT/ASCT than in those treated with dose‑dense immunochemotherapy alone (Supplementary Table 1).

**Table 3:** Response assessment

	All patients	Preplanned HDCT/ASCT	Dose-dense immuno-chemotherapy
	No. (%)	No. (%)	No (%)
Total patients	40 (100%)	23 (100%)	17 (100%)
CT	1 (2.5%)	1	-
Complete radiologic response	1	1	-
PET/CT (after completion of chemotherapy)	39 (97.5%)	22	17
PET positive*	7	0	7
PET negative*	32	22	10
CR	33 (80.6%)	23 (100%)	10 (58.8%)
PR	7 (17%)	-	7 (41.2%)
SD	0	-	-
PD/Refractory disease	1 (2.4%)		-
ORR	40 (97.6%)	23 (100%)	17 (100%)

*According to Cheson et al. JCO 2014 [14]

### Response and survival

**Fig. 1:**
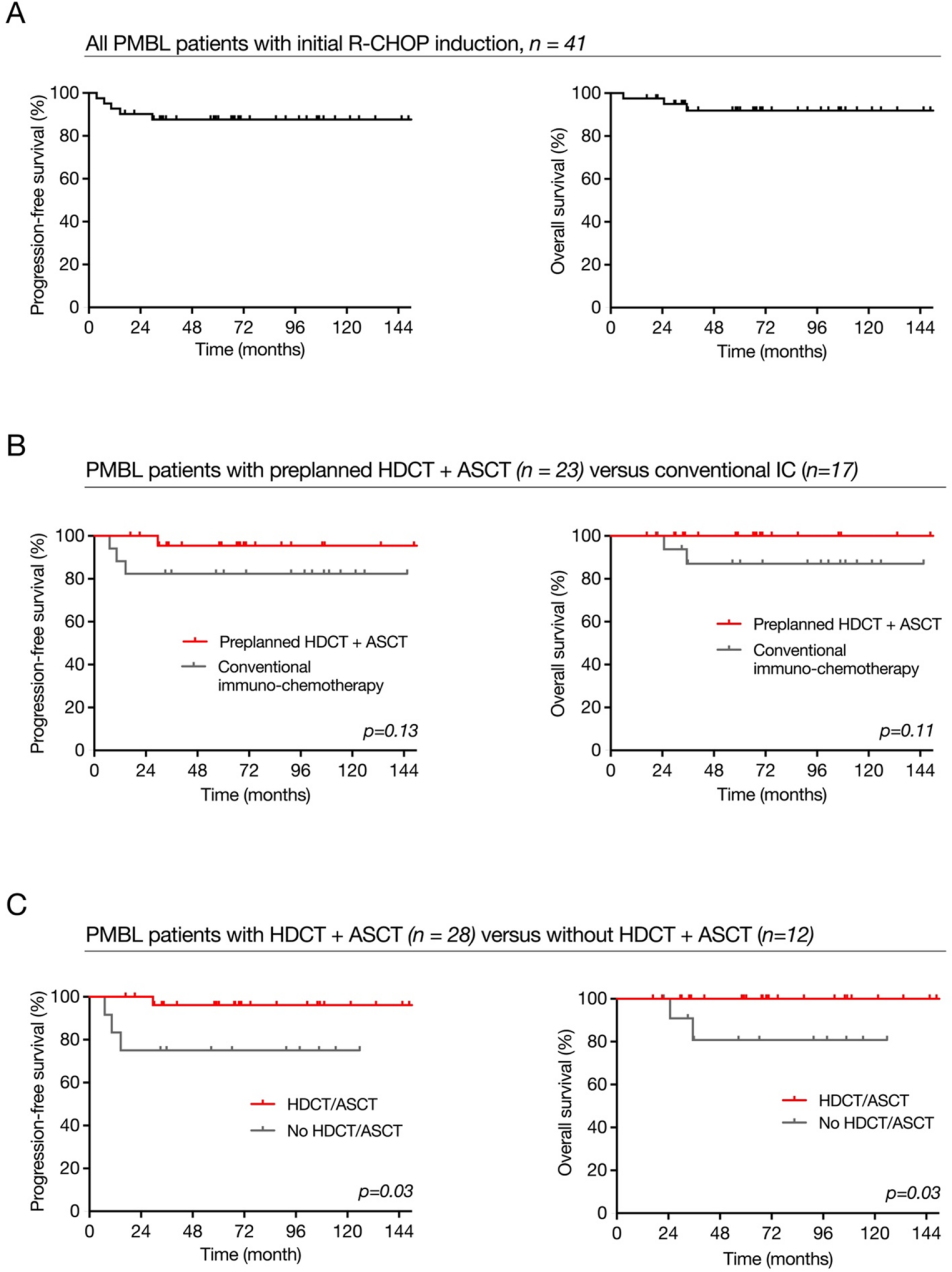
Treatment outcomes of the studied PMBL cohort. **A** Progression-free and overall survival of the entire cohort. **B** Survival data according to the preplanned consolidation strategies (Preplanned HDCT/ASCT vs. dose dense immuno-chemotherapy approach) **C** Treatment outcome with or without HDCT/ASCT consolidation, irrespective of initial consolidation strategy

The overall response rate (ORR) to first‑line therapy was 97.6%, with a complete remission rate (CRR) of 80.6%. In the planned HDCT/ASCT cohort, CRR was 100%, with all patients achieving CMR on PET/CT or complete radiologic remission on CT alone. In the dose‑dense immunochemotherapy cohort, ORR was 100% but CRR was lower at 58.8%; seven of 17 patients (41.2%) had an incomplete metabolic response (Deauville 4) after initial therapy. Detailed response data are shown in Table 3. For the entire cohort, estimated 5‑year PFS and OS were 87.7% (95% CI 72.8–94.7) and 91.9% (95% CI 76.7–97.3), respectively (Fig. 1A). Among responders (*n* = 40), 5‑year PFS and OS were 89.8% (95% CI 75.2–96) and 94.2% (95% CI 78.5–98.5). In the planned consolidation comparison (predefined HDCT/ASCT vs. dose‑dense immunochemotherapy alone), 5‑year PFS and OS were 95.5% and 100% in the planned HDCT/ASCT group versus 82.4% and 87.1% in the dose‑dense immunochemotherapy group, with a trend toward better PFS but no statistically significant difference (Fig. 1B). When patients were grouped according to whether they actually received HDCT/ASCT at any point in first‑line therapy, 5‑year PFS was 96.2% in the HDCT/ASCT group versus 75% in patients treated without HDCT/ASCT (HR 0.09, 95% CI 0.01–0.83, *p* = 0.03), and 5‑year OS was 100% versus 80.1% (HR 0.03, 95% CI 0.002-0.7, *p* = 0.03; Fig. 1C). Baseline clinical risk factors did not differ significantly between patients with and without HDCT/ASCT, although patients without HDCT/ASCT were more likely to receive RT (Supplementary Table 2). Only four relapses occurred in the entire cohort. One relapse occurred after HDCT/ASCT, and three occurred in patients initially treated with dose‑dense immunochemotherapy, two of whom had achieved initial CMR. Notably, two of these three relapses arose within previously irradiated fields. Salvage strategies included platinum‑based chemotherapy, further HDCT/ASCT and, in two cases, allogeneic transplantation. Both patients ultimately died from progressive disease or transplant‑related complications.

## Discussion

In this series of consecutively treated PMBL patients, dose-dense R-CHOP-based induction followed by HDCT/ASCT consolidation was associated with excellent long-term outcomes and a low relapse rate. Importantly, patients who underwent HDCT/ASCT received mediastinal RT significantly less often than those managed without transplant and had superior 5-year PFS and OS. These findings suggest that a transplant-based strategy may help achieve durable remission while reducing RT exposure in PMBL patients treated with dose-dense R-CHOP-based regimens.

Our results should be viewed as complementary to prospective DA‑EPOCH‑R data. In a prospective phase II study, the administration of DA-EPOCH-R resulted in equally excellent long-term outcome and RT was only applied in 4% of the patients [9]. In addition, novel front-line R-COMP-DI (liposomal doxorubicin) achieves 93% CMR in PMBL and gray zone lymphoma [18]. Whether intensified approaches without consolidation radiotherapy are the best available clinical practice is debated [19, 20]. Our institutional standard was a R‑CHOP‑14-based regimen representing a dose-dense immuno-chemotherapy approach. Multicenter data from LYSA demonstrate that R‑CHOP‑14 achieves excellent 3‑year PFS and OS (both 89.4%), comparable to R‑ACVBP and superior to R‑CHOP‑21 [21]. In a recent meta‑analysis of 4.068 PMBL patients, dose-dense regimens (including R‑CHOP‑14) were associated with improved OS/PFS and reduced RT exposure compared with R‑CHOP‑21-based therapy [22].

From a survivorship perspective, HDCT/ASCT must be weighed against its long‑term toxicity profile, particularly in young PMBL patients. Infertility in premenopausal women is a relevant issue after HDCT/ASCT. 68% premenopausal women recovered menses after BEAM conditioning [23]. After treatment with dose-adjusted EPOCH-R 74% of patients recovered menses [24]. Despite omission of RT, late toxicities of ASCT include secondary malignancies and impaired health-related quality of life [25]. HDCT/ASCT needs to be compared to intensified approaches with respect of toxicities in these patients.

In low-risk PMBL patients (aaIPI 0–1) a benefit of RT after R-CHOP was observed only in patients responding with a partial remission [26]. Despite adding mediastinal radiotherapy to the treatment, outcomes with up to 20% disease progression were reported [27–29]. In our cohort, radiotherapy rate was significantly lower in patients who received HDCT/ASCT compared to conventional immuno-chemotherapy. HDCT/ASCT treatment revealed a favorable outcome regardless of the consolidation strategy. HDCT/ASCT was generally well tolerated, however, grade 3–4 neutropenia were frequent. Primary prophylaxis with long-acting G-CSF, trimethoprim-sulfamethoxazole, aciclovir and antifungals is warranted in patients undergoing intensive regimens or ASCT. Observational data support these strategies in dose-intensified lymphomas [30] and identify immunosuppression as highest fungal infection risk [31] justifying prophylaxis [32]. Further, G-CSF-based stem cell mobilization for HDCT/ASCT can induce spleen volume increases [33]. Ultrasound methods show good correlation with CT for non-palpable splenomegaly detection [34] which are recommended for symptomatic patients.

We observed no transplant-related mortality. Our findings are in line with a retrospective analysis of the EBMT, which investigated ASCT in PMBL patients and showed an excellent long-term outcome for patients (*n* = 16) who underwent ASCT in first remission. 3-year relapse incidence was 6%; PFS and OS were 94% and 100%, respectively [35]. In the immuno-chemotherapy cohort, we observed a high rate of insufficient responses (38.9% failed CMR). The low relapse rate in our cohort likely reflects favorable disease biology and improved supportive care as much as the consolidation strategy. Since relapses are uncommon in PMBL, the high negative predictive value of EOT FDG-PET may reflect the low event rate of this disease, and one might overestimate the potential of excluding residual disease. Additionally, EOT FDG-PET has a high false positive rate [10, 36, 37] which may have contributed to overtreatment in our cohort. Results of the large randomized IELSG37 trials indicate that omission of RT is safe in patients achieving a CMR [38]. Of note, 3/4 relapses in our cohort occurred after dose-dense immuno-chemotherapy. 2/3 relapses after dose-dense immuno-chemotherapy occurred out of CMR and two relapses appeared within the radiation field.

Our study inherits the limitations of a retrospective analysis including substantial selection bias. Consolidation and radiotherapy were non‑random and physician‑driven. PET‑positive patients in the dose‑dense cohort were frequently escalated to HDCT/ASCT, leading to cross‑over of 6 patients. Together with the low number of progression events (*n* = 4), this renders the hazard ratio estimates for HDCT/ASCT versus non‑HDCT unstable. The 17‑year inclusion period inevitably introduces temporal heterogeneity; however, the R-CHOP‑14-based regimen and BEAM conditioning for ASCT remained unchanged throughout.

We studied a consecutive patient population and observed a significant better PFS and a superior OS for patients receiving HDCT/ASCT. To conclude, high-dose chemotherapy and autologous stem cell transplantation may be a valid strategy to induce a favorable long-term outcome and spare radiotherapy in patients with PMBL.

## Electronic Supplementary Material

Below is the link to the electronic supplementary material.

Supplementary Material 1 (DOCX 195 KB)

## Data Availability

The datasets used and/or analyzed during the current study are available upon reasonable request from the corresponding author.
