# Supplementary material for: Primary mediastinal B-cell lymphoma: upfront high-dose chemotherapy with autologous stem cell transplantation results in favorable long-term outcome and may spare radiotherapy
Source: Ann Hematol. 2026 Apr 17;105(5):250. doi: 10.1007/s00277-026-07009-w (PMC13090265; doi:10.1007/s00277-026-07009-w)
Supplement: Supplementary file 1 — Supplementary Material 1 (DOCX 195 KB) [file 277_2026_7009_MOESM1_ESM.docx]

**Supplementary materials**

| **Supplementary table 1** Adverse events (PR/CR patients) | | | |  |
| --- | --- | --- | --- | --- |
| Event | All patients  CTCAE Grade 3 and 4 (%) | Preplanned HDCT/ASCT  CTCAE Grade 3 and 4 (%) | Conventional immune-chemotherapy  CTCAE Grade 3 and 4 (%) | |
| Hematologic event |  |  |  | |
| Neutrophil count decreased | 35 (87.5%) | 23 (100%) | 12 (66.7%) | |
| Platelet count decreased | 28 (70%) | 22 (95.7%) | 6 (33.3%) | |
| Anemia | 26 (65%) | 19 (82.6%) | 7 (38.9%) | |
|  |  |  |  | |
| Non-hematologic event |  |  |  | |
| Acute kidney injury | 2 (5%) | 1 (4.3%) | 1 (5.6%) | |
| S-bilirubin level | 0 | 0 | 0 | |
| Elevated AST/ALT | 6 (15%) | 4 (17.4%) | 2 (11.1%) | |
| Febrile neutropenia | 26 (65%) | 19 (82.6%) | 7 (38.9%) | |
| Mucositis | 2 (5%) | 0 | 1 (5.6%) | |
|  |  |  |  | |

| **Supplementary Table 2** Results of Fisher’s exact test comparing base line variables of patients with or without HDCT/ASCT | |
| --- | --- |
| Variates | p-value |
|  |  |
| aaIPI 1-2 vs. 3 | 0.45 |
| ECOG 0-1 vs. 2 | 1.0 |
| Disease stage I/II vs. II/IV | 0.7 |
| No extranodal site vs. extranodal site involvment | 0.09 |
| Age <40y vs. >40y | 1.0 |
| Consolidation radiotherapy | 0.024 |
|  |  |

**
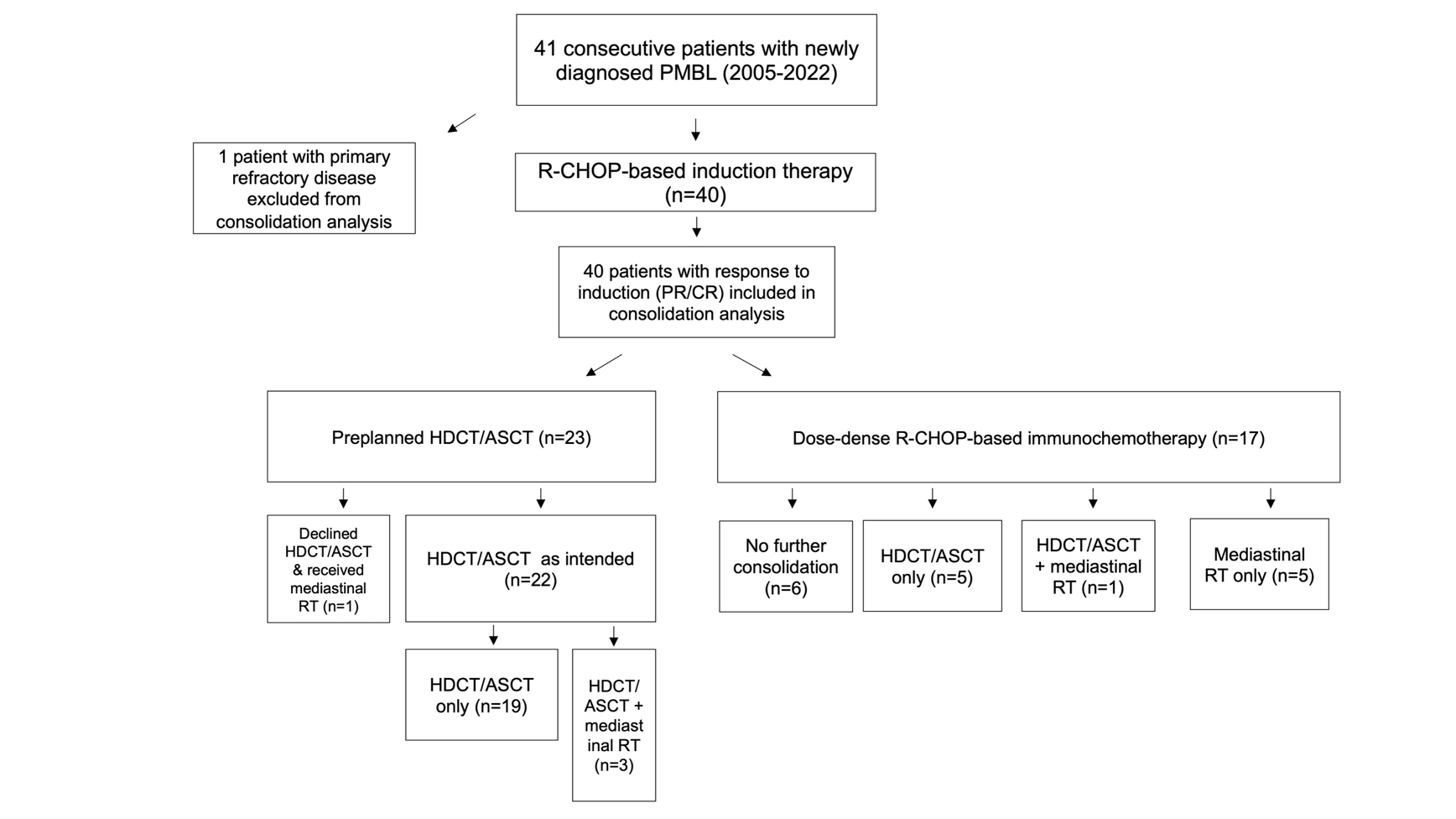
Supplementary Figure 1**

**Supplementary Methods**

The induction protocol consisted of six CHOP‑14 cycles (cyclophosphamide 750 mg/m², doxorubicin 50 mg/m², vincristine 1.4 mg/m² on day 1, prednisone 100 mg on days 1-4) combined with two cycles of high‑dose methotrexate 3 g/m² with leucovorin rescue and 10 rituximab administrations, as described previously [17]. Primary G‑CSF support (usually pegfilgrastim) and anti‑infective prophylaxis with trimethoprim‑sulfamethoxazole and aciclovir were used routinely. Full anthracycline and cyclophosphamide doses were maintained in all patients. For patients responding to induction (n=40) two consolidation strategies were carried out: Patients received either a preplanned high-dose chemotherapy (HDCT) with autologous stem cell transplantation (ASCT) (n=23) or were treated with dose-dense R-CHOP based immuno-chemotherapy only (n=17). This allocation reflects routine clinical decision‑making during the study period rather than a predefined randomization or target sample size. Stem cells were mobilized with R‑VIPE (rituximab 375 mg/m² d0; ifosfamide 4000 mg/m²; etoposide 500 mg/m²; cisplatin 50 mg/m²; cyclophosphamide 1350 mg/m²) or R-VCPE (epirubicin 50 mg/m², etoposide 500 mg/m², cisplatin 50 mg/m², cyclophosphamide 1350 mg/m²) salvage chemotherapy plus G‑CSF, and collected after 2 cycles. HDCT prior to ASCT consisted uniformly of BEAM (carmustine 300 mg/m² on day −7, etoposide 2 × 100 mg/m²/day and cytarabine 2 × 200 mg/m²/day on days −6 to −3, and melphalan 140 mg/m² on day −2), followed by autologous PBSC reinfusion on day 0. Standard transplant supportive care, including anti‑infective prophylaxis and post‑BEAM G‑CSF, was provided to all patients. No transplant‑related mortality or graft failures were observed in this cohort. In patients allocated to R-CHOP based immuno-chemotherapy only, HDCT/ASCT was performed with an insufficient response to first-line treatment, defined as failure to achieve a complete metabolic response (CMR). End‑of‑treatment FDG‑PET/CT was interpreted locally according to the Lugano/Deauville criteria; Deauville 1-3 were defined as CMR, whereas Deauville 4-5 prompted consideration of further treatment escalation and, whenever feasible, histologic confirmation. In one patient, a sarcoid‑like lesion was found on re‑biopsy despite Deauville 4 uptake, illustrating the limited specificity of EOT‑PET in fibrotic mediastinal remnants and the potential for overtreatment. Radiotherapy was administered in the discretion of the attending physician. In both cohorts, infection prophylaxis via trimethoprim/sulfamethoxazole and aciclovir was routinely given. Whenever feasible, to prevent reproductive function after chemotherapy, patients received pretreatment consultation of a reproductive specialist. Most pre-menopausal females received fertility preservation with gondatropin-releasing hormone (GnRH) analoga, e.g., goserelin acetat. Toxicities were graded with the common terminology criteria for adverse events (CTCAE) version 5.0.
